# Quantitative MRI at 7-Tesla reveals novel frontocortical myeloarchitecture anomalies in major depressive disorder

**DOI:** 10.1038/s41398-024-02976-y

**Published:** 2024-06-20

**Authors:** Jurjen Heij, Wietske van der Zwaag, Tomas Knapen, Matthan W. A. Caan, Birte Forstman, Dick J. Veltman, Guido van Wingen, Moji Aghajani

**Affiliations:** 1 Spinoza Centre for Neuroimaging, Amsterdam, The Netherlands; 2 Department of Computational Cognitive Neuroscience and Neuroimaging, NIN, Amsterdam, The Netherlands; 3 Department of Experimental and Applied Psychology, Vrije Universiteit Amsterdam, Amsterdam, The Netherlands; 4 Department of Biomedical Engineering and Physics, Amsterdam UMC, Location University of Amsterdam, Amsterdam, The Netherlands; 5 Department of Brain & Cognition, University of Amsterdam, Amsterdam, The Netherlands; 6 Department of Psychiatry, Amsterdam UMC, Location Vrije Universiteit Amsterdam, Amsterdam, The Netherlands; 7 Department of Psychiatry, Amsterdam UMC, Location University of Amsterdam, Amsterdam, The Netherlands; 8 Institute of Education and Child Studies, Section Forensic Family & Youth Care, Leiden University, Leiden, The Netherlands

**Keywords:** Depression, Diagnostic markers

## Abstract

Whereas meta-analytical data highlight abnormal frontocortical macrostructure (thickness/surface area/volume) in Major Depressive Disorder (MDD), the underlying microstructural processes remain uncharted, due to the use of conventional MRI scanners and acquisition techniques. We uniquely combined Ultra-High Field MRI at 7.0 Tesla with Quantitative Imaging to map intracortical myelin (proxied by longitudinal relaxation time T_1_) and iron concentration (proxied by transverse relaxation time T_2_*), microstructural processes deemed particularly germane to cortical macrostructure. Informed by meta-analytical evidence, we focused specifically on orbitofrontal and rostral anterior cingulate cortices among adult MDD patients (*N* = 48) and matched healthy controls (HC; *N* = 10). Analyses probed the association of MDD diagnosis and clinical profile (severity, medication use, comorbid anxiety disorders, childhood trauma) with aforementioned microstructural properties. MDD diagnosis (*p’s* < 0.05, Cohen’s *D* = 0.55–0.66) and symptom severity (*p’s* < 0.01, *r* = 0.271–0.267) both related to decreased intracortical myelination (higher T_1_ values) within the lateral orbitofrontal cortex, a region tightly coupled to processing negative affect and feelings of sadness in MDD. No relations were found with local iron concentrations. These findings allow uniquely fine-grained insights on frontocortical microstructure in MDD, and cautiously point to intracortical demyelination as a possible driver of macroscale cortical disintegrity in MDD.

## Introduction

Brain abnormalities are increasingly postulated in Major Depressive Disorder (MDD) [1]. Although a complete understanding of its underlying neuropathology is still lacking, perturbed cortical gray matter morphology is emerging as a fairly consistent correlate of MDD [1–4]. Structural MRI studies pinpoint the prefrontal and anterior cingulate cortices as the main site of these perturbations [1–4], consistent with their hub-like function within canonical brain networks governing salience processing, affective responding, and complex decision-making [5–13]. Massive meta-analytical examinations within the ENIGMA MDD Consortium specifically highlight cortical thinning of orbitofrontal and rostral anterior cingulate cortices as fairly robust correlates of MDD [4]. However, as in-vivo *macrostructural* estimates such as gray matter thickness/surface area/volume only quantify shape changes of inner and outer cortical boundaries [14, 15], we still lack insight into *microstructural* processes occurring within the cortical mantle among MDD patients.

Among microstructural processes ostensibly germane to cortical morphology, intracortical myelination and local iron concentration have emerged as promising candidates [16–18]. Intracortical myelination is found predominately in the deeper cortical layers, and considered critical for establishing and maintaining neural circuits and functional networks [16, 18–21], processes deemed partly affected in MDD [1, 22]. Intracortical myelin is highly sensitive to environmental processes, contributing heavily to experience-dependent neural plasticity and remodeling across the lifespan [20, 21]. Preliminary neuroimaging data and small postmortem studies cautiously suggest a role for intracortical demyelination, specifically within prefrontal regions, in the etiology of clinical depression [23–26]. Changes in intracortical myelin content are moreover believed to partly mediate above-mentioned cortical disintegrity in MDD, as neural fibers mostly start from or end in cortical gray matter [27], with intracortical demyelination accordingly being coupled to diminished cortical volumes [28–30]. Optimal iron concentration is an essential element required for oligodendrocytes to synthesize myelin across the lifespan. However, excessive iron levels not only prevent remyelination, but can also induce oxidative stress, free radical toxicity, and eventually neural cell death via various cellular pathways [16–18, 31, 32]. Imbalanced iron concentrations further upset human neurophysiology (e.g., neurotransmitter synthesis and metabolism, oxygen transport, neural transmission), neurocognition (e.g., executive function, attention, memory) and social behavior (e.g., isolation, wariness, withdrawal), with increased brain iron deposits being tentatively linked to depression [33–35].

While mapping intracortical myelin and iron concentration clearly carries relevance for fine-grained microstructural understanding of cortical disintegrity in MDD, use of conventional MRI scanners and acquisition techniques have so far precluded truly robust examinations. Prior work has almost-exclusively employed standard field strength MRI (1.5/3.0 Tesla), with typical spatial resolutions of 1 mm. As the human cortex is approximately 2–4 mm thick, this standard 1 mm resolution is unable to accurately map intracortical myelin or iron content in different cortical layers [16, 18, 36]. Ultra-high field (UHF) MRI at 7.0 Tesla and above now allows for sub-millimeter in-vivo examination of the brain, permitting depth-dependent cortical investigations that are unprecedently fine-grained and consistent with ex-vivo work [16, 18, 36]. The combination of UHF MRI with modern quantitative acquisition techniques, so-called quantitative MRI, has emerged as particularly powerful in microstructural mapping of distinct cortical layers [16, 18, 36]. Unlike conventional MRI, quantitative MRI provides specific biophysical measures of microstructural integrity within the brain (e.g., intracortical myelin, axons, glia, iron), which are comparable across brain regions, populations, and scanners, by employing so-called relaxation parameters [16, 17]. This brings great advantages for clinical research, as a ‘normative’ baseline can be set and compared to patient data [37], even with fairly small normative control groups. The longitudinal relaxation time T_1_ serves as an inverse proxy for myelin content [16], while the apparent transverse relaxation time T_2_* is a proxy for local iron concentration [16, 38] (see “Methods” for detailed technical description).

Despite its noteworthy potential, no study has yet capitalized on the complementary use of UHF MRI and quantitative imaging for fine-grained microstructure mapping of cortical tissue in MDD. Detailed quantification of the extent, type, and spatial distribution of cortical tissue anomalies in MDD could provide novel cellular insights into disease pathomechanisms, which ultimately might serve as putative biomarkers and therapeutic targets. This study, hence, uniquely employed UHF MRI (7.0 Tesla) and quantitative imaging to map intracortical myelin and iron concentration in MDD patients. We focused specifically on the orbitofrontal and rostral anterior cingulate cortices, as massive meta-analytical data specifically highlight neurostructural anomalies within these regions as a fairly robust correlate of MDD [4]. Based on prior work, we anticipated abnormal intracortical myelin and iron concentration in these regions, both as a function of MDD diagnosis and clinical profile (severity/antidepressant medication/comorbid anxiety/childhood trauma).

## Methods

### Participants

Seventy-three individuals were recruited for this study. After exclusion of 15 participants (Supplementary Information; Data Exclusion), the final sample consisted of 58 individuals, consisting of 48 MDD patients and 10 healthy control (HC) participants. Inclusion criteria for MDD patients were: primary DSM-5 diagnosis of current MDD (6 months recency) as determined by the Composite International Diagnostic Interview (CIDI) [39], and referred to mental health care. Inclusion criteria for HC participants were: no history of depression diagnosis or treatment, nor any other psychopathology, and normal or subclinical scores on dimensional measures of psychopathology.

Exclusion criteria for the entire sample were: (1) presence of psychoses, mania, Tourette’s syndrome, or obsessive-compulsive disorder; (2) diagnosis of major internal or neurological disorders; (3) traumatic head injury; (4) current substance abuse or dependence requiring treatment; (5) evidence of acute suicidal risk requiring immediate intervention; (6) MRI contraindications, including metal implants, heart arrhythmia, or claustrophobia; (7) left-handedness; (8) pregnancy; (9) inadequate understanding of the Dutch language, (10) aged <20 and >55 years. Ethical review board of the Amsterdam UMC (location VUmc) approved this study, and written informed consent was obtained from all participants. Our study was conducted in accordance with the declaration of Helsinki, with all performed methods being in line with relevant guidelines and regulations. Detailed sample characteristics are provided in Table 1.

**Table 1:** Characteristics of the sample.

Characteristic	MDD (*N* = 48)	HC (*N* = 10)
Age (years)^ns^	36.53 ± 10.63 (20.92–55.63)	38.84 ± 8.13 (27.28–49.52)
Sex (*N*, %)^ns^		
Female	35 (73%)	6 (60%)
Male	13 (27%)	4 (40%)
Education (years)^ns^	14.59 ± 3.02 (10–18)	15.09 ± 1.45 (15–18)
IDS^a^	33.47 ± 13.53 (9–64)	4.1 ± 3.07 (0–7)
CTQ^ns^	46.77 ± 19.18 (26–98)	39.9 ± 9.24 (25–54)
Age of onset	21.72 ± 10.99 (5–49)	–
Psychotropic medication (*N*, %)	27 (56.3%)	
–Antidepressants	24 (50.0%)	–
–Benzodiazepines	6 (12.5%)	–
–Antipsychotics	5 (10.4%)	–
Comorbid anxiety disorder (*N*, %)	21 (43.8%)	–
–Social anxiety	7 (14.6%)	–
–Generalized anxiety	2 (4.2%)	–
–Panic disorder	4 (8.3%)	–
–Mixed	8 (16.7%)	–

Categorical variables (sex) were tested using Chi-square test, whereas continuous variables were tested with ANOVA. Means ± standard deviations, and ranges (in parentheses) are provided for quantitative measures. 1 MDD patient did not complete the IDS and CTQ.

*MDD* major depressive disorder, *HC* healthy controls, *IDS* inventory of depressive symptomatology, *CTQ* Childhood Trauma Questionnaire, *SSRI* selective serotonin reuptake inhibitors.

^ns^Not significant at *p* < 0.05.

^a^Significant at *p* < 0.001.

Due to the financial burden and time constraints at play when conducting UHF MRI research, we chose to maximize and prioritize the inclusion of our main target group (MDD), which resulted in a relatively small HC sample. As mentioned earlier though, quantitative MRI partly safeguards against and compensates for such unbalanced sample sizes, as a “normative” baseline can be set and compared to patient data, even with a fairly small control group [37]. The groups were matched for age, sex, and education. The Inventory of Depressive Symptomatology (IDS [40]) and Childhood Trauma Questionnaire (CTQ [41]) were used to evaluate depressive symptom severity and childhood trauma, respectively.

### MRI data acquisition

Images were acquired at the Spinoza Centre for Neuroimaging in Amsterdam, the Netherlands using a Philips Achieva 7.0 Tesla MRI scanner equipped with a 32-channel head array coil (Nova Medical). T_1_-maps and T_2_*maps were obtained simultaneously using an MP2RAGEME (multi-echo magnetization-prepared rapid gradient echo) sequence [42]. The MP2RAGEME is an extension of the MP2RAGE sequence [43] and consists of two rapid gradient echo (GRE_1,2_) images that are acquired after a 180° degrees inversion pulse and excitation pulses with inversion times TI_1,2_ = [670 ms, 3675.4 ms]. A multi-echo readout was used in the second inversion, with four equally spaced echo times (TE_1_ = 3 ms, TE_2,1–4_ = 3, 11.5, 19, 28.5 ms). Other scan parameters include flip angles FA_1,2_ = [4°,4°]; TR_GRE1,2_ = [6.2 ms, 31 ms]; bandwidth = 404.9 MHz; TR_MP2RAGEME_ = 6778 ms; acceleration factor SENSE_PA_ = 2; FOV = 205 × 205 × 164 mm; acquired voxel size = 0.7 × 0.7 × 0.7 mm; acquisition matrix was 292 × 290; reconstructed voxel size 0.64 × 0.64 × 0.7 mm; turbo factor (TFE) = 150, resulting in 176 shots; Total acquisition time = 19.53 min. A similar protocol has been used previously in cohort studies [44]. Acquisition of fat navigators were interleaved with the MP2RAGEME sequence to perform motion correction [45], thereby improving edge definition [46].

The signal of the MP2RAGEME can be analytically described based on the inversion, echo and repetition times [42]. T_1_-maps were computed using a look-up table from this model with the sequence parameter values [42, 44]. As previously mentioned, T_2_* relaxation refers to the transversal decay of magnetization after excitation due to local field inhomogeneities induced by tissue or other materials [47]. GRE-based sequences are sensitive to these inhomogeneities, as tissue and materials cause faster dephasing and thus signal loss [47]. This gradual dephasing of the signal intensity is sampled by the MP2RAGEME sequence using the multi-echo readout scheme during the GRE-block. A least-squares fitting of the exponential signal decay over the multi-echo images of the second inversion resulted in the T_2_*-maps [42].

T_1_-weighted imaging relies on the longitudinal spin-lattice relaxation for contrast [48, 49]. This contrast shows myelin-sensitivity as demonstrated by its robust white/gray matter contrast. The mechanistic relationship between faster longitudinal relaxation as measured by T_1_ and an increased myelin concentration is thought to relate to increased spin-polarization exchanges between the water-protons bound to the lipid macromolecules of the myelin sheath and the water protons of the intra/extracellular spaces [50–53]. As such, T_1_ measurements have found wide usage as a non-invasive proxy measure of myelin content [54–58]. T_1_-contrast correlates well with myelin immunohistochemistry [57, 59], and is highly stable across subjects and scan protocols [54].

T_2_* is the relaxation time associated with the combined effect of spin-spin interaction and (microscopic) magnetic field inhomogeneity on transverse magnetization [60]. One element that influences the microscopic magnetic field is iron [61], the most abundant paramagnetic trace element [62]. The higher the local iron concentration, the faster the dephasing (T_2_*) [63]. This is why iron-rich structures appear hypointense on T_2_*-weighted MRI [64–66]. Within the laminae of the cortex, the T_2_*-contrast seems to be driven by the ferritin stored in intracortical fibers [67, 68].

### Data analysis

#### Regions of interest

After preprocessing of the anatomical images (Supplementary Information; MRI Data Processing and Fig. S1), we selected three regions-of-interest from the Desikan-Killiany atlas: the rostral anterior cingulate cortex (rACC), medial orbitofrontal cortex (mOFC), and lateral orbitofrontal cortex (lOFC). As mentioned earlier, massive meta-analytical examinations within the ENIGMA MDD Consortium specifically highlight cortical thinning in these regions as robust correlates of MDD (largest effect sizes) [4]. These ROIs were sampled to each subject’s individual volumetric space. Because these ROIs are defined in FreeSurfer space (and can therefore still be suboptimal around superficial layers), we dilated the masks with 1–3 voxels and multiplied this with the optimized gray matter segmentation, yielding optimized FreeSurfer ROI masks. These masks were then applied to the T_1_-/T_2_*-maps to sample quantitative relaxation time values from 10 cortical depths using the *profile sampling* module from Nighres [69], resulting in 10 values for each hemisphere of each region for each subject. The profile sampling module leans on the laminar module, which in turn benefited greatly from our segmentation strategy (Fig. S1). This ensured that we could sample superficial layers more accurately than a strategy solely based on FreeSurfer.

#### Statistical inferences

We used JASP [70] for statistical inference, which included both case-control and within-patients analyses to thoroughly examine the impact of MDD on cortical myeloarchitecture. Similar to previous studies [25], average T_1_ (inverse proxy for myelin concentration) and T_2_* (inverse proxy for iron concentration) were assessed across ROIs as global measure. This metric averages all T_1_/T_2_* values across cortical depths and is blind for the specific distribution across the cortical ribbon. Global, then, refers to the ROI-level. Utilizing the high resolution of the acquisition, we assessed T_1_/T_2_-values at 10 depths parameterized as area-under-curve (AUC; depth-dependent measure) from a 3rd-order polynomial fit to the T_1_/T_2_* points across depth, providing an indication of myelin/iron concentration within the ROIs. While this metric still consists of 1 value, it is more sensitive to the distribution of T_1_/T_2_*-values across depth [71]. This metric belongs to the family cortical myelin features that capture subtle (non-linear) layer-specific changes related to development and pathology [14, 36, 72, 73] and complements traditional volumetric myelin measures. Lastly, we assessed T_1_/T_2_* at the WM/GM transitional zone (by means of the intercept parameter from the polynomial fit), an important transitional zone from white to gray matter that ostensibly signifies connectional strength between white and gray matter [74].

Because testing relaxation times across cortical depth is severely hampered by the multiple comparisons problem, we opted for more parsimonious metrics such as area-under-curve and shape estimation using polynomial fits. Analyses of covariance (ANCOVA) compared MDD and HC patients on these metrics, while controlling for between-groups variation in age and sex. For regions where significant group differences emerged, additional exploratory analyses within patients probed whether MDD severity, antidepressant use, comorbid anxiety disorders, and childhood trauma were linked to more severe alterations in above-mentioned metrics. In line with recent ROI-based intracortical myelin examinations in psychiatric populations [25, 75, 76], significance level was set at alpha = 0.05, though additionally complemented with effect size and confidence interval estimates, so to ascertain precision of findings and balance Type I and II error rates [77, 78].

#### Global effects analyses

To exclude the possibility that parenchymal changes in T_1_ values were related to global volumetric differences, we ran a Voxel-Based Morphometry (FSL-VBM [79, 80]) analysis using our optimized segmentations by adapting the standard VBM workflow. Instead of brain extraction and segmentation as implemented in the first step of VBM, the optimized gray matter segmentations (Fig. S1, *combined*) were formatted to be compatible with the next step of VBM; the images were averaged and flipped along the *x*-axis to create a left-right symmetric, study-specific gray matter template. To maintain resolution, we did not perform smoothing. Finally, voxel-wise general linear modeling (GLM) was applied using permutation-based non-parametric testing, correcting for multiple comparisons across space.

## Results

### Sample characteristics

As shown in Table 1, MDD and HC groups did not differ on age, sex, and education. MDD patients scored higher on depression severity, while no between-group differences were found for childhood trauma index. Majority of MDD patients were using antidepressants (SSRI) and presented comorbid anxiety disorders.

### Average T_1_ and T_2_* maps

**Fig. 1:**
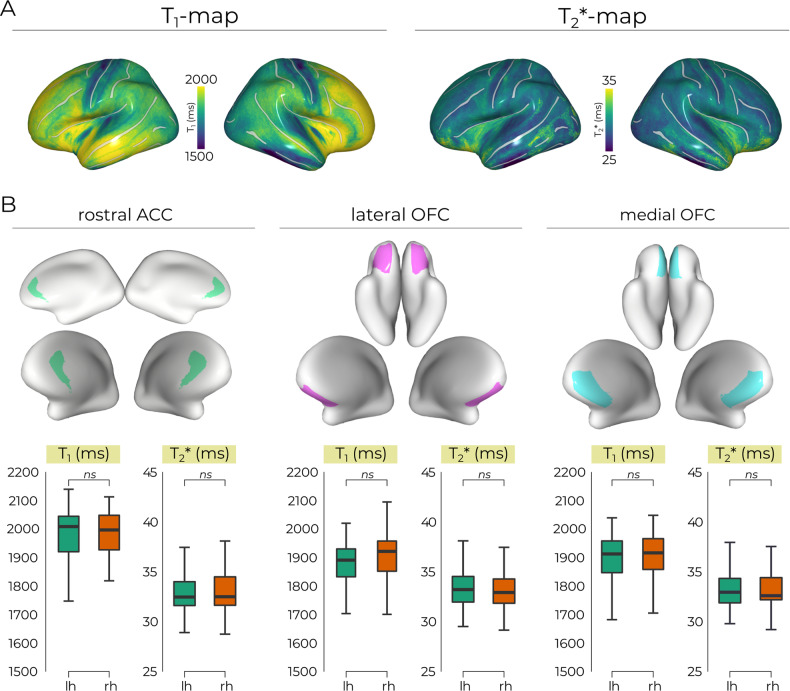
Data fidelity confirmation. **A** Classical pattern of reduced T_1_-values (inverse proxy for myelin concentration) in the sensorimotor cortex across hemispheres, with standard T_2_* values (proxy for iron concentration) typically reported with 7.0 Tesla MRI. **B** Average T_1_-/T_2_*-values across hemispheres for each specific ROI, which showed no statistical differences (*p* > 0.05). rACC rostral anterior cingulate cortex, mOFC medial orbitofrontal cortex, lOFC lateral orbitofrontal cortex, lh left hemisphere, rh right hemisphere, ns not significant at *p* > 0.05.

To confirm data fidelity, we assessed general characteristics of the quantitative MRI data by sampling the T_1_-and T_2_*-maps to the surface (*FSAverage*). Figure 1A shows the classical pattern of reduced T_1_-values (inverse proxy for myelin concentration) in the sensorimotor cortex [81–84]. T_2_* values (inverse proxy of iron concentration) were also within the range generally reported at 7T [42, 85, 86]. After this sanity check, we zoomed in on our regions-of-interest and assessed lateralization of T_1_/T_2_*-values, but did not observe statistically significant differences across hemispheres (*ps* > 0.05) (Fig. 1B). The presented results from here on therefore represent data averaged across hemispheres to further limit multiple comparisons.

### MDD diagnosis relates to intracortical myelin—but not iron—concentrations

**Fig. 2:**
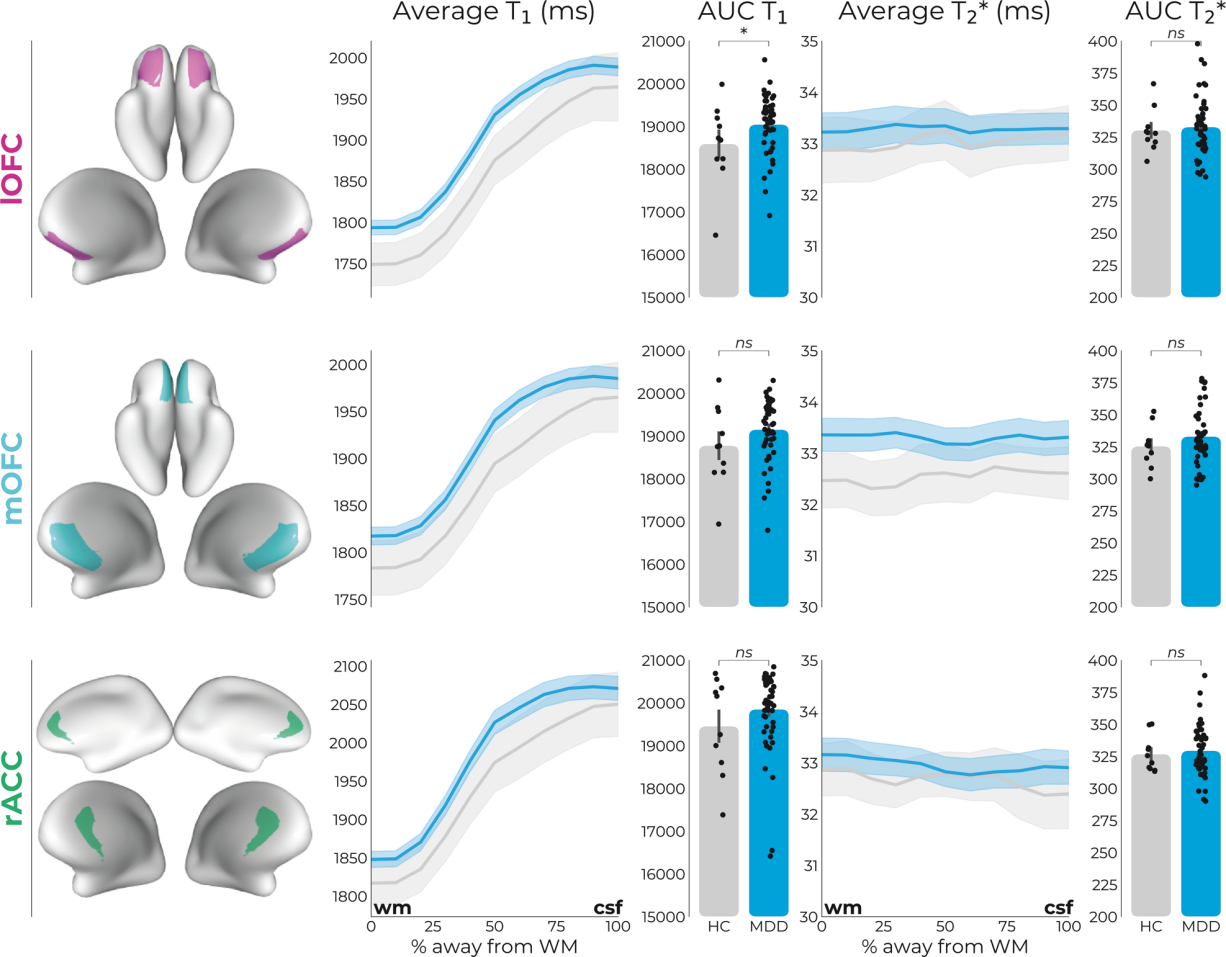
MDD diagnosis relates to intracortical myelin—but not iron—concentrations. MDD patients showed decreased intracortical myelin concentration (higher average T_1_ values), within the lOFC. MDD patients also showed depth-dependent changes in lOFC, as evidenced by higher T_1_-AUC values (distribution of myelin across 10 cortical depths). No case-control differences were found for either average (T_2_*) or depth-dependent (AUC T_2_*) iron concentration levels. rACC rostral anterior cingulate cortex, mOFC medial orbitofrontal cortex, lOFC lateral orbitofrontal cortex, WM white matter, CSF cerebrospinal fluid, ns not significant at *p* > 0.05, * = significant at *p* < 0.05.

**Fig. 3:**
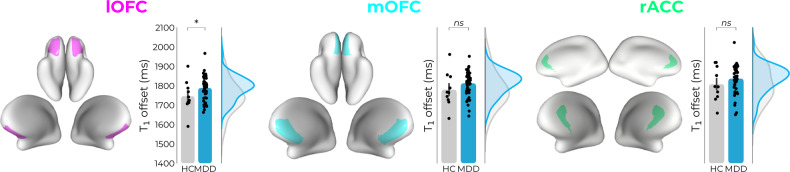
MDD diagnosis relates to intracortical myelin changes in WM/GM transitional zone. MDD patients showed decreased base levels of intracortical myelin in WM/GM transitional zone (higher T_1_ offset values) within the lOFC region. rACC rostral anterior cingulate cortex, mOFC medial orbitofrontal cortex, lOFC lateral orbitofrontal cortex, WM white matter, CSF cerebrospinal fluid, ns not significant at *p* > 0.05, * = significant at *p* < 0.05.

For all ROIs, the T_1_-profiles (inverse proxy for myelin concentration) of MDD patients lie above those of the HC participants (Fig. 2; line plots). However, only in the lOFC this effect was also statistically significant (*F*_1,54_ = 4.174, *p* = 0.046, Cohen’s *D* = 0.55, CI = −85.55 to −0.807), with higher average T_1_-values in MDD (*M* = 1903.36, SD = 67.52) compared to HC (*M* = 1858.89, SD = 98.89). In the lOFC, we also found a slight depth-dependent change in shape of the profile (*F*_1,54_ = 4.369, *p* = 0.041, Cohen’s *D* = 0.60, CI = −856.82 to −18.04), as evidenced by higher T_1_-AUC (distribution of T_1_ across 10 cortical depths) in MDD (*M* = 19,045.75, SD = 677.24) compared to HC (*M* = 18,590.97, SD = 956.38). To formally test the upwards shift of average T_1_ profiles in MDD vs. HC, we probed the T_1_ offset parameter (Fig. 3), which provides an inverse proxy for myelin concentration at the WM/GM transitional zone by fitting a 3rd-order polynomial function to the data points. This revealed a significant effect of diagnostic status on the base levels of intracortical myelin in the lOFC (*F*_1,54_ = 4.722, *p* = 0.034, Cohen’s *D* = 0.66, CI = −80.31 to −3.23), in which MDD patients showed higher T_1_ near the WM/GM transitional zone (*M* = 1788.83, SD = 59.73) compared to HC (*M* = 1746.40, SD = 81.23).

Of note, we limited the possibility that these effects were due to global effects, by additionally probing the primary somatosensory cortex (S1); a region that is not considered to play a key role in MDD psychopathology. As anticipated, no major effects were found for this region (*p’s* > 0.05, Fig. S2). Additionally, we tested whether differences were driven by gray matter volumes using VBM analysis or altered T_1_-values in underlying white matter (Fig. S3), which was not the case (*p’s* > 0.05). These T_1_-value (i.e., inverse proxy for intracortical myelin) changes thus collectively pinpoint decreased intracortical myelin concentration within lOFC among MDD patients relative to HC participants. As depicted in Fig. 2, no other case-control effects were found for T_1_-values, neither did any case-control effects emerge for T_2_*-parameters (iron concentration) (*p’s* > 0.05).

### MDD severity relates intracortical myelin

**Fig. 4:**
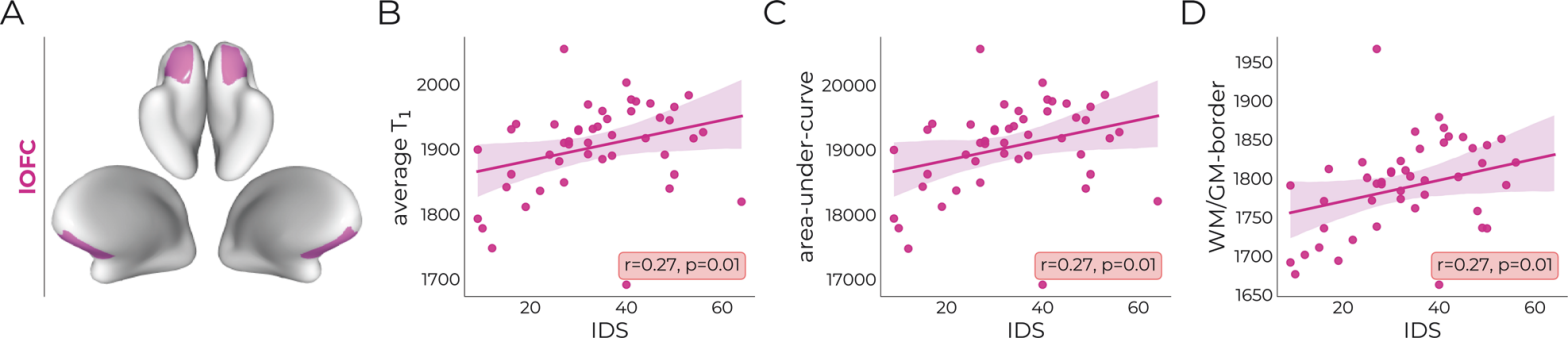
MDD severity relates to intracortical myelin concentrations in lOFC. MDD severity (IDS score) relates to increases in lOFC average T_1_ (inverse proxy for myelin concentration, panel **B**), T_1_ AUC (distribution of T_1_ across 10 cortical depths, panel **C**), and T_1_ offset (WM/GM transitional zone, panel **D**). Correlations (Kendall’s *τ*) and associated *p*-values for total IDS score in relation to T_1_-profile metrics within the lOFC are shown in red boxes. Panel **A** depicts the lOFC region of interest. lOFC lateral orbitofrontal cortex, WM white matter, GM gray matter, IDS inventory of depressive symptomatology.

Additional analyses across patients probed whether clinical features (severity, antidepressant use, comorbid anxiety disorders, childhood trauma) had any impact on MDD-related changes in lOFC intracortical myelin. The analyses showed that higher MDD severity (IDS score) related to more pronounced increases in average T_1_ (inverse proxy for myelin concentration; *r* = 0.269, *p* = 0.008, CI = 0.054–0.485), T_1_ AUC (distribution of T_1_ across 10 cortical depths; *r* = 0.271, *p* = 0.008, CI = 0.056–0.486), and T_1_ offset (T_1_ at WM/GM transitional zone; *r* = 0.267, *p* = 0.008, CI = 0.048–0.487), indicative of diminished intracortical myelin concentration (Fig. 4). No effects were found for antidepressant use, comorbid anxiety disorders, and childhood trauma (*p’s* > 0.05, see Fig. S4).

## Discussion

This study harnessed the power of UHF quantitative MRI for the very first time for assessing microstructural processes that may putatively underlie previously reported anomalies in cortical macrostructure in MDD. We focused specifically on intracortical myelin and iron concentration within orbitofrontal and rostral anterior cingulate cortices, regions meta-analytically linked to MDD pathophysiology. Largely in line with our hypotheses, MDD diagnosis and symptom severity both related to decreased intracortical myelin concentration (higher T_1_ values) within the lOFC, a region tightly coupled to processing negative affect and feelings of sadness in MDD. These findings allow uniquely fine-grained insights on frontocortical microstructure in MDD, and cautiously point to intracortical demyelination as a possible driver of macroscale cortical disintegrity in MDD.

### MDD diagnosis and severity relate to intracortical myelin changes

MDD patients showed decreased intracortical myelin concentration (higher T_1_ values), within the lOFC, a region deemed particularly relevant in the pathophysiology of depression [4, 87]. This decrease in lOFC intracortical myelin additionally related to more severe MDD symptoms, further underscoring its relevance to symptomatic manifestation of MDD. These diagnostic and severity-related decreases were seen across intracortical depths (average T_1_), but also at depth-dependent level (AUC) and for myelin at the WM/GM transitional zone (intercept parameter of 3rd-order polynomial fit) (see Figs. 2–4). Of note, we did not find any MDD-related changes in iron concentration levels (T_2_* values) within frontal and cingulate regions that were examined. This might speculatively suggest that intracortical myelin and its underlying microstructural processes are ostensibly more germane than iron concentration vis-à-vis cortical disintegrity in MDD, a notion that warrants further investigation and future replication. Intracortical myelination is found predominately in the deeper cortical layers, and considered critical for establishing and maintaining neural circuits and functional networks [16, 18–21], processes deemed partly affected in MDD [1, 22]. Converging lines of evidence highlight abnormal intracortical myelination in the onset of psychopathology, as intracortical myelination levels and onset of psychiatric disorders such as MDD both tend to peak during early and middle adulthood (20–35 years) [88, 89]. Animal data importantly shows that changes in intracortical myelination typically precede shifts in neurofunctional and behavioral patterns, underscoring the putative cascading effects of demyelination on maladaptive behavior [90, 91].

While biological mechanisms underlying abnormal myelin content in MDD are not yet fully understood, decreased axonal activity and stress-induced neuroinflammation have emerged as putative causal factors [25, 92]. Myelination is an adaptive process dependent on environmental influences through axonal firing rates [92], and data does link heightened axonal firing to increased myelination [92, 93]. As such, optogenetic stimulation of the rodent cortex was shown to increase local myelination [94, 95], while drug-induced myelination of frontal cortex in rodents co-occurs with diminished depressive behaviors [96]. Stress-induced neuroinflammation is deemed another possible driver of demyelination in MDD, wherein prolonged/chronic stress sets off excessive release of proinflammatory cytokines, which in turn can affect and damage the structural integrity of myelin [97, 98]. In support of this notion, elevated proinflammatory cytokines were linked to demyelination of cerebellar tissue [99]. While we lacked the data to directly scrutinize these putative pathomechanistic pathways, future research should examine how intracortical myelin content in MDD patients may map onto cortical axon activity and circulating cytokines levels over time.

### lOFC primary effect site intracortical myelin changes

Decreased intracortical myelination documented here in relation to MDD diagnosis and severity was restricted to the lOFC, a region often linked to depression [4, 87]. In fact, massive meta-analytical examinations within the ENIGMA MDD Consortium (*N* > 10,000) specifically highlight cortical thinning within the lOFC region as a fairly robust correlate of MDD [4]. However, as in-vivo macrostructural estimates such as cortical thickness only quantify shape changes of inner and outer cortical boundaries [14, 15], microstructural processes occurring within the cortical mantle among MDD patients remain fairly uncharted. Leveraging UHF quantitative MRI, we cautiously point to intracortical demyelination as potentially relevant in MDD-related cortical thinning of the lOFC. While this notion warrants cautious interpretation and further investigation, preliminary human neuroimaging and postmortem data link intracortical demyelination to MDD pathophysiology [23–26], with intracortical demyelination partly mediating cortical macrostructure disintegrity [27, 29, 88, 100].

The emergence of the lOFC as primary site of MDD-related intracortical demyelination is particularly intriguing, as structural, functional, and connectional disintegrity of this region is increasingly linked to hypersensitivity for negative affective stimuli in MDD [87]. It is postulated that a negative affect network is centered in the lOFC, with rich reciprocal connections to fronto-cingulo-limbic and temporo-parietal territories [87], whose network function mainly serves domain-general negative affect processing and saliency [87]. In MDD patients, the lOFC typically shows local hyperresponsivity and negative affect network hyperconnectivity when faced with punishing, unpleasant, and non-rewarding information, which apparently fuels negative self-image and feelings of chronic sadness [87]. Recent data even shows that lOFC hyperresponsivity to punishment or non-rewarding events (i.e., absence/decrease of anticipated rewards) maps onto severity of depressive symptoms [101]. It is also reported that transcranial magnetic stimulation of the lOFC tends to normalize its activity, and diminish depression in a substantial proportion of patients [102, 103]. Along the same lines, successful treatment with antidepressant medication seemingly coincides with normalized activity and functional connectivity patterns of the lOFC-centered negative affect network in MDD patients [87, 104]. Given the importance of intracortical myelin for establishing and maintaining neural circuits and functional networks [16, 18–21], future work should examine how successful MDD treatment maps onto intracortical myelination and associated circuit/network-level function.

### Limitations

The modest sample size and cross-sectional nature of this study do not allow for causal inferences about the myeloarchitecture changes documented here in MDD, rendering it difficult to exactly pinpoint what may precede or follow them at the neurobiological level. The cross-sectional design also precludes examination of the progressive impact that MDD illness duration might exert on intracortical myelin content. Longitudinal studies can elegantly address all these issues, but also provide insights into the relationships intracortical myelination may showcase with risk of relapse, long-term medication use, and treatment efficacy in MDD. Replication of ENIGMA MDD findings regarding cortical thickness/surface area/volume was not one of our aims, and frankly rather impossible to achieve given disparities in sample size (*N* = 58 vs. *N* > 10000) and data type (7.0 Tesla vs. 1.5/3.0 Tesla). Likewise, reaffirming previously established relationships between cortical microstructure and macrostructure was not achievable here, and was therefore not an aim. These relationships are rather subtle and intricate, involving many other processes beyond just myeline/iron concentrations, with some of them difficult to capture even at 7.0 Tesla resolution. We also lacked the sample size and statistical power to effectively capture such subtle/intricate interdependencies. Future UHF MRI studies in significantly larger samples, comprising a broader set of cortical microstructure features (beyond just myelin/iron), are warranted to tackle these limitations and address frontocortical microstructure-macrostructure relationships in MDD.

Due to the financial burden and time constraints at play when conducting UHF MRI research, we chose to maximize and prioritize the inclusion of our main target group (MDD), which resulted in a relatively small HC sample. Yet, the use of quantitative MRI may partly safeguard against and compensate for these unbalanced sample sizes. Unlike conventional MRI, quantitative MRI provides specific biophysical measures of microstructural integrity within the brain, which are comparable across brain regions, populations, and scanners, by employing so-called relaxation parameters [16, 17]. This brings great advantages for clinical research, as a “normative” baseline can be set and compared to patient data [37], allowing robust case-control examinations even with a fairly small control group. That said, the 48 MDD patients included here render this the largest UHF MRI examination of brain myelin/iron concentrations in MDD to date. Our modest overall sample size may have plausibly also played a role in the null findings on local iron concentrations (T_2_*), as a larger sample may have potentially improved statistical power, and thus detection of ostensibly more subtle changes in iron concentration. Despite these limitations, our findings allow uniquely fine-grained insights on frontocortical microstructure disintegrity in MDD, whilst also serving as an important point of departure for future studies.

## Conclusions

This study harnessed the power of UHF quantitative MRI for the very first time for assessing microstructural processes that may putatively underlie previously established anomalies in cortical macrostructure in MDD. Results showed that MDD diagnosis and symptom severity both relate to decreased intracortical myelin concentration (higher T_1_ values) within the lOFC, a region tightly coupled to processing negative affect and feelings of sadness in MDD. These findings allow uniquely fine-grained insights on frontocortical microstructure in MDD, and cautiously point to intracortical demyelination as a possible driver of macroscale cortical disintegrity in MDD. Detailed quantification of the extent, type, and spatial distribution of cortical tissue anomalies in MDD as implemented here, could provide unprecedented cellular insights into disease pathomechanisms that ultimately might serve as biomarkers and therapeutic targets. Future UHF MRI studies should thus investigate how altered intracortical myelination maps onto MDD clinical features, and whether these alterations can be corrected or prevented with treatment.

### Supplementary information

SUPPLEMENTAL MATERIAL

## Data Availability

Data from this article are not publicly available because of limitations in ethical approval. Some summary data might be available upon reasonable request directed to the corresponding author.
