# Supplementary material for: Quantitative MRI at 7-Tesla reveals novel frontocortical myeloarchitecture anomalies in major depressive disorder
Source: Transl Psychiatry. 2024 Jun 20;14:262. doi: 10.1038/s41398-024-02976-y (PMC11190139; doi:10.1038/s41398-024-02976-y)
Supplement: Supplementary file 1 — SUPPLEMENTAL MATERIAL [file 41398_2024_2976_MOESM1_ESM.docx]

***Supplementary information***

**Supplementary Methods**

**MRI Data Processing**

First, a spatial-adaptive Non-Local Means (*SANLM*-) filter implemented in CAT12 (<https://neuro-jena.github.io/cat/>) was applied to the T1w-images to filter noise while maintaining edges [1]. The denoised image was segmented into cerebrospinal fluid (CSF), white matter (WM) and gray matter (GM) using CAT12 and corrected for intensity non-uniformity with *N4BiasFieldCorrection* [2], distributed with ANTs 2.3.3. A mask representing the sagittal sinus was by subtracting the brain mask from CAT12 from the brain mask based on the second inversion (INV2) image from the MP2RAGEME acquisition[3]. The voxels in the mask were set to zero in the denoised T1w image to reduce manual intervention after surface reconstruction. The final masked image was then used as input for the structural preprocessing module of fMRIprep[4], where the image was skull-stripped with a Nipype implementation of the *antsBrainExtraction.sh* workflow[5], using OASIS30ANTs as target template. FreeSurfer 7.2 *recon-all*[6] was used to obtain native cortical surface reconstructions for each participant. After surface reconstruction, segmentations derived from FreeSurfer[6], CAT12, and manual edits were averaged and used as input for cortical reconstruction using the *CRUISE* algorithm[7] as implemented in Nighres[8] (Figure S1). However, ROI segmentations were executed in *FreeSurfer*-space, based on the white matter and pial boundaries. To maximally benefit from the optimized GM segmentation, we sampled the ROIs to the subjects’ volumetric space, dilated them by ~2 voxels, and multiplied that with the optimized GM segmentation. This resulted in more accurate coverage of superficial layers.

**Data exclusion**

Data of 15 participants (11 patients) did not pass the quality assessment due to poor data quality, segmentations still missing superficial regions of the ROIs due to similarities between CSF/background and GM, or missing data, resulting in a final sample size of 58 (48 patients).

**Supplementary Figures**


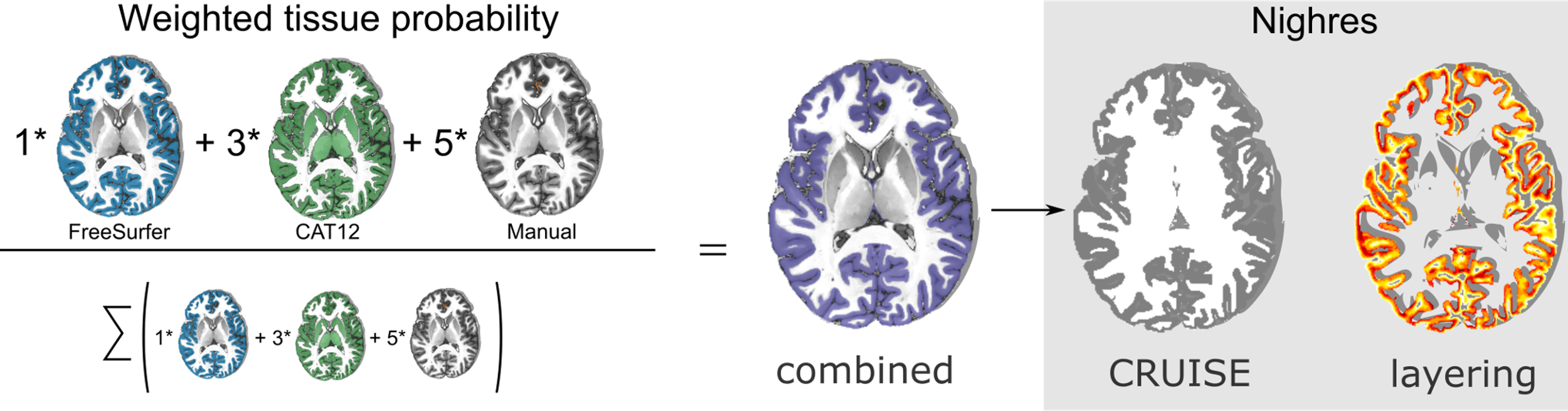


Figure S1. To improve segmentation quality, outputs from FreeSurfer and CAT12 were combined with manual edits (if needed) to create custom weighted probability maps for gray matter, white matter, and CSF. These optimized probability maps were then fed into the CRUISE and volumetric layering module from Nighres. T_1_-and T_2_*-values were then sampled across depth using Nighres’ profile sampling module, which leans on the volumetric layering output. Therefore, an improved segmentation will result in more accurate sampling across depth.

**

Figure S2. Global effects were ruled out by including a control region, in this case the primary somatosensory cortex. Here, no effects are observed in the profiles and offsets of the MDD group compared to the HC group. ns = not significant at p<0.05

Figure S3. T_1_ values in the white matter mask, showing that parenchymal T_1_ changes in cortex were not related to white matter differences across groups (t_56_ = -0.699, p=0.49). ns = not significant at p<0.05

Figure S4. Within-patients comparisons of T_1_-profiles based on various clinical characteristics did not reveal any significant differences. Green profiles represent the absence of a feature (e.g., use of antidepressant/psychotropic medication, childhood trauma, and comorbid anxiety).

**References**

1. Manjón JV, Coupé P, Martí-Bonmatí L, Collins DL, Robles M. Adaptive non-local means denoising of MR images with spatially varying noise levels. Journal of Magnetic Resonance Imaging. 2010;31:192–203.

2. Tustison NJ, Avants BB, Cook PA, Zheng Y, Egan A, Yushkevich PA, et al. N4ITK: Improved N3 Bias Correction. IEEE Transactions on Medical Imaging. 2010;29:1310–1320.

3. Caan MWA, Bazin P-L, Marques JP, de Hollander G, Dumoulin SO, van der Zwaag W. MP2RAGEME: T(1) , T(2)(*) , and QSM mapping in one sequence at 7 tesla. Human Brain Mapping. 2019;40:1786–1798.

4. Esteban O, Markiewicz CJ, Blair RW, Moodie CA, Isik AI, Erramuzpe A, et al. fMRIPrep: a robust preprocessing pipeline for functional MRI. Nat Methods. 2019;16:111–116.

5. Avants BB, Epstein CL, Grossman M, Gee JC. Symmetric diffeomorphic image registration with cross-correlation: Evaluating automated labeling of elderly and neurodegenerative brain. Medical Image Analysis. 2008;12:26–41.

6. Dale AM, Fischl B, Sereno MI. Cortical Surface-Based Analysis: I. Segmentation and Surface Reconstruction. NeuroImage. 1999;9:179–194.

7. Han X, Pham DL, Tosun D, Rettmann ME, Xu C, Prince JL. CRUISE: Cortical reconstruction using implicit surface evolution. NeuroImage. 2004;23:997–1012.

8. Huntenburg JM, Steele CJ, Bazin P-L. Nighres: processing tools for high-resolution neuroimaging. GigaScience. 2018;7.
